# Progress in Medicine

**Published:** 1897-04-03

**Authors:** 


### Progress in Medicine.

### RHEUMATISM AND GOUT.

The affections, which are by many authorities said to be connected with the poison of rheumatism, include purpura, angeioneurotic œdema, chorea, and several others. It is not improbable, however, that some of them have no such relation, and that many cases called rheumatism are really instances of distinct diseases. Thus there seem to be types of purpura which are superficially like those found in attacks of rheumatism, but which differ essentially. A. Ernest Peake[1] records a case of Henoch's purpura in a woman aged 23. There was no personal or family history of rheumatism. [It should, however, be noticed that these rheumatic histories when present are often most misleading, and refer to previous attacks of something else.] She had first an erythematous rash, pains in the knees, but without swelling, and muscular pains. Then followed abdominal pains, hæmatemesis and melæna with great prostration, and, lastly, a profuse purpuric rash on the skin and mucous membrane of the mouth. The joints of the arms were very painful, but no albumen or other kidney complication occurred, and the patient made a good recovery. L. Weber[2] had a case of purpura hæmorrhagica in a glycosuric patient, following some indiscretion in his food. There had previously been an attack, with rheumatoid pains, six years before. In another instance there had been several attacks of gonorrhœa, and in consequence of exposure during the purpura the patient developed hæmorrhagic pneumothorax. In a discussion which followed the report of the cases, others were narrated showing the infectious character of the disease. Sternberg, indeed mentions that Letzerich infected himself by his pipe while examining the bacteria discovered in a patient, and suffered from a severe attack. There is no doubt some difficulty in distinguishing between simple purpura occurring in numerous dyscrasia and drug poisonings, and the hæmorrhagic form, which is probably a microbic infection. A recent discovery of Bensaude[3] goes far to settle this question. In ordinary blood, if withdrawn from the vessels, the clot, when formed, contracts and squeezes out the serum, but in p. hæmorrhagica this does not happen, while there is a marked decrease in the number of hæmatoblasts. These two marks were found in 16 cases of various types of the disease, including two where it was concurrent with phthisis. All of them showed extensive cutaneous echymoses and mucous hæmorrhages. In the simple purpura of rheumatism, of toxic and cachetic states these two symptoms were not present. The changes were best marked before or during the attacks of bleeding, and can be produced in animals, together with symptoms of purpura by inoculation of the blood of patients affected with the disease. Thus there are two very different states, included under the name of purpura, the one a definite microbic infection, and the other a mere symptom found in various diseases; but the former may occur either alone or may complicate other disorders, such as phthisis.

**Angeioneurotic Œdema** is comparatively rare, and has only been discussed in recent years, though isolated cases have been long on record. Circumscribed local swellings appear in various parts of the body, and may be associated with gastric pain and disturbance. All ages and both sexes are affected, and the swellings may resemble gigantic wheals or loose œdematous masses of varying size, which tend to recur again and again There is an hereditary factor in some of the instances; but R. Norton, who gives a very full account of the affection,[4] remarks that the exciting causes are very numerous and ill-defined. The body temperature is not usually affected, and the œdema is never inflammatory or dependent on position. Some burning or pricking may precede the swelling, and at times there is some itching where it is present. Two patients died from œdema of the glottis, but generally the attacks are repeated frequently, sometimes daily, without any disturbance of the health. The urine is said to be diminished during the attacks, and there is occasionally slight albuminuria. Many of the patients suffer from rheumatoid arthritis, but usually the œdemas are seen near joints where no arthritis exists. It should be diagnosed from urticaria, hysterical, and syphilitic. œdema, as well as that met with in kidney and heart disease and in the neighbourhood of abscesses.

Rheumatism in newly-born children is rarely discovered. Cheadle only mentions two cases, but Abrahams[5] has recently reported three where the mothers suffered from acute attacks at or before delivery. In one the symptoms appeared at birth, but pus formed in one knee with a fatal result. Syphilis could be excluded, but the mother had just recovered from acute rheumatism. The mother of the second child died of rheumatic hyperpyrexia after labour, and the child showed typical symptoms at birth and recovered under salicylates, the temperature having once reached 103·5 deg. The third child was born after its mother had gone through rheumatic fever and chorea, and though it showed no joint symptoms, aortic insufficiency was present. Endocarditis existed in both the other children.

[1] B. Med. Jour., Jan. 2, 1897. [2] New York Med. Rec., Jan. 9. [3] Med. Week, Jan. 22. [4] New York Med. Jour., Jan. 30. [5] Med Record, Oct 17.

